# Editorial: Improving Diagnosis, Treatment, and Prognosis of Neuropsychiatric Disorders by Leveraging Neuroimaging-based Machine Learning

**DOI:** 10.3389/fnins.2022.891337

**Published:** 2022-04-13

**Authors:** Baojuan Li, Hongbing Lu, Yu-Feng Zang, Hui Shen, Qiuyun Fan, Jian Liu

**Affiliations:** ^1^School of Biomedical Engineering, Fourth Military Medical University, Xi'an, China; ^2^Institutes of Psychological Sciences, Hangzhou Normal University, Hangzhou, China; ^3^College of Intelligence Science and Technology, National University of Defense Technology, Changsha, China; ^4^Department of Biomedical Engineering, College of Precision Instruments and Optoelectronics Engineering, Tianjin University, Tianjin, China; ^5^Network Center, Fourth Military Medical University, Xi'an, China

**Keywords:** machine learning, neuroimaging, neuropsychiatric disorders, diagnosis, treatment, prognosis

Recently, there has been increasing attempt that leveraged machine learning techniques to improve the diagnosis, treatment, and prognosis of diseases with medical images. Pioneering work has consistently demonstrated the promise of machine learning in a variety of clinical settings, such as detection of diabetic retinopathy using retinal fundus photographs (Gulshan et al., 2016), identification of axillary lymph node metastasis with magnetic resonance imaging (MRI) radiomics in patients with breast cancer (Yu et al., 2020), prediction of the risk of patients' sudden cardiac death with MRI and positron-emission tomography (PET) images (Shade et al., 2021), etc. The employment of machine learning techniques in these clinical settings has substantially advanced the development of computer-assisted systems for disease diagnosis and treatment.

Machine learning has also demonstrated its great potential in the diagnosis and treatment of neuropsychiatric disorders. Many of these diseases are now redefined as “disorders of brain circuits” (Insel et al., 2010). As for mental disorders, traditional diagnosis of these disorders is merely based on subjective symptoms and signs. In 2010, the National Institute of Mental Health (NIMH) launched the Research Domain Criteria (RDoC) project, which aims to establish a framework for classification of mental disorders based on underlying biological mechanisms with data from genetics and clinical neuroscience (Insel et al., 2010). This initiative has changed our understanding of mental disorders and may hopefully promote “precision medicine for psychiatry” (Insel et al., 2010; Insel and Cuthbert, 2015). Indeed, recent studies have shown the feasibility of redefining and subtyping of mental disorders according to patterns of brain activity and connectivity obtained from neuroimaging data with the help machine learning. For example, Drysdale and the colleagues identified four neurophysiological subtypes of depression according to patients' functional connectivity profiles with hierarchical clustering (Drysdale et al., 2017). Later, Li et al. reported that functional striatal abnormalities could be used to reliably identify schizophrenia patients with a high accuracy, as well as predict patients' response to antipsychotic treatment (Li et al., 2020).

This Research Topic aims to advance our understanding of healthy and diseased brains by leveraging neuroimaging-based machine learning, in order to improve diagnosis, treatment, and prognosis of neuropsychiatric disorders. We thus invited world-renowned experts to present their recent work that have employed novel machine learning approaches in studies of neuropsychiatric disorders. Of the 25 papers published in this Research Topic, the majority focused on the diagnosis of neuropsychiatric disorders. Due to the relatively small sample size of the datasets, support vector machine (SVM) was the most widely used machine learning method in these papers. Noninvasive neuroimaging data, including structural and functional MRI images, electroencephalography and magnetoencephalography were collected and the classifiers were generally trained and tested with features generated by a traditional morphological analysis or brain connectivity analysis. Now with the help of machine learning techniques, we are capable of detecting subtle and complex abnormalities in brain structure and function in patients with neuropsychiatric disorders. It is worth noting that the functional connectivity profiles are now considered to constitute a fingerprint of the brain which could be used to reliably differentiate a subject from others (Finn et al., 2015).

We will see in this Research Topic that Pan et al. achieved an accuracy of higher than 80% in differentiating patients with cervical dystonia and healthy controls, using voxel-wise resting-state functional connectivity and SVM. Similar framework was used to identify patients with Parkinson's disease, type 2 diabetes mellitus induced cognitive impairment, major depression, obsessive-compulsive disorder, bipolar disorder, internet addiction, as well as High-Risk First-Degree Relatives of Patients With Schizophrenia. In addition, Zhao et al. established a novel pipeline for epileptogenic zone identification with convolutional neural network. Kung et al. showed that morphological features could be employed to identify the conversion from mild cognitive impairment to Alzheimer's disease with multilayer perceptron classifier. Moreover, Inglese et al. established a self-supervised contrastive learning model for subtyping of patient with systemic lupus erythematosus.

In addition to disease diagnosis, This Research Topic also includes novel and interesting findings on the application of machine learning in treatment and prognosis of diseases. Li et al. showed that neuroimaging features could be used to predict response to treatment in patients with idiopathic generalized epilepsy with tonic–clonic seizures. Xi et al. showed that brain structure-based signature could be used to identify responders and non-responders to a treatment with combined ECT and antipsychotics in patients with schizophrenia. Bohaterewicz et al. proposed a framework to predict suicide risk in patients with schizophrenia. Xu et al. established a nomogram model for the prediction of intracerebral hematoma expansion with radiomic features extracted from CT images.

Looking into the future, the reproducibility of the findings and the generalization of the classifiers may need to be tested with large datasets from multiple independent sites. The sample size in most of the papers published in this Research topic is relatively small and whether findings could be reproduced with large datasets remains to be tested. More importantly, the majority of studies in this Research Topic have trained and tested the model with data from a single site. Whether these models could still achieve high classification accuracy when tested with data collected from another independent site remains unclear. Unfortunately, due to the high heterogeneity of neuropsychiatric disorders, machine learning models built on the data from a single site usually demonstrated poor performances when tested with data from multiple independent sites. To ensure the robustness and generalization of the classifiers, future studies may need to further train the model with multi-site dataset and systematically tested the generalization of the models with leave-one-site-out cross validation.

## Author Contributions

BL and JL wrote the first draft of the manuscript. HL, Y-FZ, HS, and QF revsied the manuscript. All authors contributed to the article and approved the submitted version.

## Funding

This work was supported by the National Natural Science Foundation of China (61976248, 82071994), and Hunan Innovative Province Construction Special Project (2020SK2097).

## Conflict of Interest

The authors declare that the research was conducted in the absence of any commercial or financial relationships that could be construed as a potential conflict of interest.

## Publisher's Note

All claims expressed in this article are solely those of the authors and do not necessarily represent those of their affiliated organizations, or those of the publisher, the editors and the reviewers. Any product that may be evaluated in this article, or claim that may be made by its manufacturer, is not guaranteed or endorsed by the publisher.
